# 

**Published:** 1848-10

**Authors:** 


### JOURNAL.

ARTICLE I.—Report on Practical Dentistry, at the Eighth Annual Meeting of the Am. Soc. of Dental Surgeons. By C. O. Cone, D. D. S., . . . . . . . . . . . . . 3

ARTICLE II.—Letter from Dr. A. Hill, Norwalk, Ct., . . . . 82

ARTICLE III.— Cases of Tic Douloureux successfully treated with Lunar Caustic. By Dr. S. P. Hullihen, . . . . . 86

ARTICLE IV.—Decay in Teeth. By J. Lee, M. D., of Camden, S. C., 96

ARTICLE V.—Perforation of the Antrum Maxillare at the Base of the Malar Process. By W. H. Dwinelle, D. D. S., . . . . 103

ARTICLE VI.—A Case of Abscess of the Antrum Highmorianum, and Caries of the Outer Wall. By J. Weatherby, Dentist, . 106

ARTICLE VII.—Observations on Hemorrhage. By B. B. Brown, M. D., D. D. S., of St. Louis, Mo., . . . . . . 108

ARTICLE VIII.—Proceedings of the Society of Dental Surgeons of the State of New York, . . . . . . . . . 124

ARTICLE IX.—Report of the Proceedings of the Pennsylvania Society of Dental Surgeons, . . . . . . . . . 128

### COLLECTANEA.

On the action of Atrophine in Painful Affections of the Face, . . . 133

On Exostoses: their Character, . . . . . . . . . 133

Foreign Body lodged in the Cheek for ten months, . . . . . 134

Case of Necrosis and Expulsion of the Os Hyoides, . . . . 135

Cancrum Oris, . . . . . . . . . . . . . 135

Cases of Affection of Upper Jaw, . . . . . . . . . 136

Cures for the Cholera, . . . . . . . . . . . 138

Progress of the Cholera, . . . . . . . . . . 139

Lawrence's Improved Portable Blow-Pipe, . . . . . . 141

Composition of a Salivary Calculus taken from a Horse, . . . 142

Diagnosis of Hypertrophy of the Brain from Chronic Hydrocephalus, 142

### MISCELLANEOUS NOTICES.

Ninth Annual Meeting of the American Society of Dental Surgeons, 143

Practical Hints, . . . . . . . . . . . . . 147

The Library Part of our Journal, . . . . . . . . 151

Dr. Elliot's File Carrier, . . . . . . . . . . 152

Cholera, . . . . . . . . . . . . . . . 152

To our Readers, . . . . . . . . . . . . . 153

Agents of the Journal, . . . . . . . . . . . 153

